# Combination of Photodynamic Therapy and a Flagellin-Adjuvanted Cancer Vaccine Potentiated the Anti-PD-1-Mediated Melanoma Suppression

**DOI:** 10.3390/cells9112432

**Published:** 2020-11-07

**Authors:** Hye Suk Hwang, Kondareddy Cherukula, Yong Jun Bang, Veena Vijayan, Myeong Ju Moon, Jayalakshmi Thiruppathi, Sao Puth, Yong Yeon Jeong, In-Kyu Park, Shee Eun Lee, Joon Haeng Rhee

**Affiliations:** 1 Department of Microbiology, Chonnam National University Medical School, Hwasun-gun, Jeonnam 58128, Korea; hshwang33@gmail.com (H.S.H.); biopandemic@naver.com (Y.J.B.); jayasheshavst123@gmail.com (J.T.); puthsao55@gmail.com (S.P.); 2 Clinical Vaccine R&D Center, Chonnam National University, Hwasun-gun, Jeonnam 58128, Korea; 3 Combinatorial Tumor Immunotherapy MRC, Chonnam National University Medical School, Hwasun-gun, Jeonnam 58128, Korea; 4 Department of Biomedical Sciences, Chonnam National University Medical School, Hwasun-gun, Jeonnam 58128, Korea; cherrikonda@gmail.com (K.C.); veenavj4392@gmail.com (V.V.); 5 Department of Radiology, Biomolecular Theranostics (BiT) Laboratory, Chonnam National University Medical School, Hwasun-gun, Jeonnam 58128, Korea; mjmoon2398@gmail.com (M.J.M.); yjeong@jnu.ac.kr (Y.Y.J.); 6 Department of Pharmacology and Dental Therapeutics, School of Dentistry, Chonnam National University, Gwangju 61186, Korea

**Keywords:** photodynamic therapy, FlaB-adjuvanted peptide vaccine, PD-1 blockade, B16-F10 melanoma, combination therapy

## Abstract

Immune checkpoint inhibitors become a standard therapy for malignant melanoma. As immune checkpoint inhibitor monotherapies proved to have limited efficacy in significant portion of patients, it is envisaged that combination with other therapeutic modalities may improve clinical outcomes. We investigated the effect of combining photodynamic therapy (PDT) and TLR5 agonist flagellin-adjuvanted tumor-specific peptide vaccination (FlaB-Vax) on the promotion of PD-1 blockade-mediated melanoma suppression using a mouse B16-F10 implantation model. Using a bilateral mouse melanoma cancer model, we evaluated the potentiation of PD-1 blockade by the combination of peritumoral FlaB-Vax delivery and PDT tumor ablation. A photosensitizing agent, pheophorbide A (PhA), was used for laser-triggered photodynamic destruction of the primary tumor. The effect of combination therapy in conjunction with PD-1 blockade was evaluated for tumor growth and survival. The effector cytokines that promote the activation of CD8^+^ T cells and antigen-presenting cells in tumor tissue and tumor-draining lymph nodes (TDLNs) were also assayed. PDT and FlaB-Vax combination therapy induced efficacious systemic antitumor immune responses for local and abscopal tumor control, with a significant increase in tumor-infiltrating effector memory CD8^+^ T cells and systemic IFNγ secretion. The combination of PDT and FlaB-Vax also enhanced the infiltration of tumor antigen-reactive CD8^+^ T cells and the accumulation of migratory CXCL10-secreting CD103^+^ dendritic cells (DCs) presumably contributing to tumor antigen cross-presentation in the tumor microenvironment (TME). The CD8^+^ T-cell-dependent therapeutic benefits of PDT combined with FlaB-Vax was significantly enhanced by a PD-1-targeting checkpoint inhibitor therapy. Conclusively, the combination of FlaB-Vax with PDT-mediated tumor ablation would serve a safe and feasible combinatorial therapy for enhancing PD-1 blockade treatment of malignant melanoma.

## 1. Introduction

Melanoma is the most aggressive and invasive form of skin cancer and can metastasize to virtually any organ of the body: it has very low survival rate and easy relapse tendency. Although modern targeted therapies such as BRAF inhibitors are showing some promise, mutations in *BRAF* are observed in approximately 50% of skin melanomas and are linked to acquired resistance, which occurs in half of diagnosed patients [1]. The success of immune checkpoint inhibitors (ICIs) in melanoma treatment drastically changed the therapeutic landscape of not only the intractable later stage melanomas but also other malignancies [2,3,4]. The photodynamic therapy (PDT), due to its minimally invasive characteristics and mild side effects (normal tissue preservation, relatively less pain, and bleeding tendency compared with other regimens), represents a promising alternative treatment for primary lesions of melanomas [5]. When activated by harmless light source, the photosensitizers function as catalysts upon light absorption and then convert molecular oxygen to reactive oxygen species (ROS), which induce tumor cell death and vascular shutdown [6,7]. PDT has been shown to release tumor antigens and immunogenic damage-associated molecular patterns (DAMPs) from affected tumor cells [8]. By combining these immunologic effects, PDT creates a favorable microenvironment for tumor antigen expansion and antigen-presenting cell activation [9]. However, PDT is hard to be applied to metastatic lesions at distant organ sites [10]. Any immunotherapeutic modality that would take advantage of PDT-induced immunogenic cell death and tumor microenvironment (TME) modulation mediated by released DAMPs should be able to activate potent immune responses that could suppress distantly metastasized tumor cells. Moreover, the PDT-mediated TME modulation should create significantly “hotter” immunological niche where ICIs and tumor killing immune cells will become more active.

Regarding ICIs, several agents have become the standard care drugs through numerous clinical trials with outcome improvements in recurrent and/or metastatic melanoma [4,11,12,13,14]. However, patients with certain neoantigens expressed only in a subset of their tumor cells (subclonal neoantigens) appeared to respond poorly to checkpoint blockade [15]. In melanoma cancer treatments, the response to pembrolizumab is associated with a higher number of CD8^+^, PD-1^+^, and PD-L1^+^ cells within tumor tissue, suggesting the need for reinvigorating pre-existing T cells in the tumor by inhibiting the PD-1/PD-L1 signaling cascade in TME [16]. Moreover, since PD-1 blockade in unprimed or suboptimally primed CD8^+^ cells rather induces resistance, timely cancer vaccine combination is suggested as an obliging option for breaking the resistance [17]. The patients who responded to pembrolizumab had increased frequencies of tumor-infiltrating CD8^+^ memory T cells compared to those of nonresponders [18]. Any physicochemical and immunotherapeutic approaches that would facilitate infiltration of tumor killing immune cells will further improve the therapeutic efficacy of ICI treatments.

For successful cancer immunotherapy, systemic immunity should be sufficiently activated to fight against metastases and prevent recurrence. It has been well demonstrated that cancer vaccines employing tumor-associated antigens (TAAs) can induce substantial tumor-specific immunities and epitope expansion [19], which should be an advantage over other modalities enhancing pre-existing immunity nonspecifically, such as ICI or cytokine therapies [20]. Moreover, vaccines loaded with multiple peptides can be used to activate multiple T cell clones reactive against diverse epitopes and to offer a long-term immune memory preventing cancer recurrence [21]. TAA vaccines, being generally less immunogenic than neoantigen vaccines, need appropriate adjuvants to achieve clinically satisfactory efficacy. In our previous studies, we have shown that the TLR5 agonist flagellin served as an excellent adjuvant inducing effective cell-mediated immunity (CMI) against coadministered TAA peptide epitopes [22,23] and flagellin-secreting bacteria modulated TME to induce effective antitumor immune response [24].

To address all the issues raised above, we herein propose a combinatorial ICI therapeutic modality strategically employing PDT and flagellin-adjuvanted tumor-specific peptide vaccination (FlaB-Vax). The combination was cooperative in inducing tumor-specific CMI suppressing tumors in a bilateral mouse B16-F10 melanoma model, which is classified as immunologically “cold” [25]. The combination of peritumoral FlaB-Vax delivery with PDT effectively induced a systemic and local response of peptide tumor antigen-specific IFNγ-secretions and accumulation of effector memory CD8^+^ T cells, which further enhanced PD-1 blockade therapeutic outcome. Compared with PDT alone, the combination regimen released a higher amount of CXCL10 cytokines that would contribute to more effective CD8^+^ T cell infiltration and antigen cross-presentation by CD103^+^CD11C^+^ DC subsets.

## 2. Methods

### 2.1. Cells

B16-F10 cells were purchased from ATCC and cultured in complete Dulbecco’s modified Eagle’s medium (DMEM, Gibco; supplemented with 10% heat-inactivated FBS, 100 units/mL penicillin, 100 μg/mL streptomycin, and 4 mM l-alanyl-l-glutamine). Single cells from spleen or tumor-draining lymph nodes (inguinal lymph nodes, TDLNs) were cultured in RPMI supplemented with 10% heat-inactivated FBS, 100 units/mL penicillin, and 100 μg/mL streptomycin. Cells were cultured at 37 °C and 5% CO_2_.

### 2.2. Synthesis of Liposome-Pheophorbide A (Lipo-PhA) and Photodynamic Therapy (PDT)

**Figure 1:**
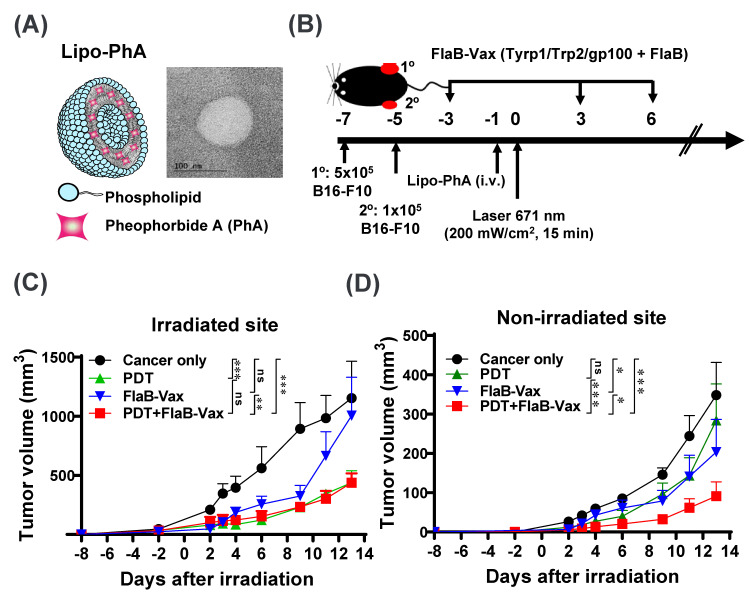
Inhibition of the growth of distant tumors in mice treated with photodynamic therapy (PDT) and flagellin-adjuvanted tumor-specific peptide vaccination (FlaB-Vax) at the primary tumor site. (**A**) Schematic illustration of pheophorbide A-loaded liposomes. Field-emission transmission electron microscopy (FE-TEM) images of liposome. (**B**) Schema of the PDT and FlaB-Vax combination therapy protocol. Growth of irradiated primary (**C**) and nonirradiated abscopal tumors (**D**) in mice treated with PDT, FlaB-Vax, or PDT + FlaB-Vax (*n* = 5 per group). Asterisks indicate *p* values for the comparison of each group in irradiated tumors or nonirradiated tumors by two-way ANOVA. *, *p* < 0.05; **, *p* < 0.01; ***, *p* < 0.001.

Liposome-based pheophorbide A photosensitizer (Lipo-PhA) was synthesized by the thin-film hydration method [26], followed by extrusion (Figure 1A). Briefly, dipalmitoyl-sn-glycero-3-phosphocholine (DPPC; Avanti polar lipid, Alabama, USA), 1,2-distearoyl-sn-glycero-3-phosphoethanolamine-N-(methoxy (polyethylene glycol)-2000) (DSPE-PEG; Avanti Polar Lipid, Alabama, USA), cholesterol lipids (Avanti Polar Lipid, Alabama, USA), and PhA (Frontier Scientific Inc., Logan, USA) at 1.5:1.5:1:0.5 weight ratio were combined in a chloroform/methanol mixture and subjected to evaporation to form a thin lipid film. Next, the lipid film was hydrated in 1 mL of phosphate-buffered saline (PBS) for 30 min at 60 °C in the dark to form heterogeneous and multivesicular liposomes. The mixture was vortexed and sonicated under ice for 7 min, and the obtained solution was passed through a 200 nm polycarbonate filter fixed in an Avanti miniextruder (Avanti Polar Lipids, Alabama, USA) for 11 cycles to obtain Lipo-PhA. The unloaded PhA was removed by 100 kDa centrifugal filters (Merck Millipore, Co., County Cork Ireland). The nanoparticle size (DLS analysis) and zeta potential were assessed using a Zetasizer Nano Z instrument (Malvern Instruments, Malvern, UK). The morphology of the nanoparticles was analyzed by field-emission transmission electron microscopy (FE-TEM) (JEM-2100F JEOL, Tokyo, Japan), and the UV absorbance was analyzed using a UV–VIS spectrophotometer (UV-2700Shimadzu, Tokyo, Japan).

Tumors were treated 7 days after B16-F10 tumor implantation at an average tumor diameter of 5 mm. First, 5 mg/kg of Lipo-PhA was intravenously injected via tail vein at -1 day; 24 h later, the tumors were irradiated with a 674 nm Milon Lakhta laser. A continuous irradiation protocol of 15 min at 200 mW/cm^2^ was used, which was decided after pilot optimization experiments. For irradiation, the skin in the tumor area was shaved, and the mice were anesthetized and positioned horizontally on a heat pad. Precise irradiation of the tumor was ensured by fixing the laser emitting fiber optic vertically above the mouse and adjusting the exposed area using a diaphragm.

### 2.3. Peptide Antigen and FlaB Adjuvant for Cancer Vaccine

The peptide antigen sequences are as follows: Trp2_180–188_ (SVYDFFVWL), Tyrp1opt_455–463_ (optimized A463M57, TAPDNLGYM), and gp100opt_20–39_ (optimized S27P, EGP long58, AVGALEGPRNQDWLGVPRQL) (Anygen, Gwangju, Korea) [27]. *V. vulnificus* FlaB was prepared as previously described [28]. For the production of FlaB protein, a 1.5-kb fragment containing the open reading frame of *V. vulnificus* FlaB was cloned into the pTYB12 yielding pCMM250 vector (New England Biolabs). For the purification of recombinant FlaB, the bacterial pellet was resuspended in a lysis buffer (20 mM Tris-Cl (pH 7.5), 500 mM NaCl, 1 mM EDTA (pH 8.0), 0.1% Triton X-100, 0.1% Tween 20, 20 μM phenylmethylsulfonyl fluoride) and sonicated (Vibra Cell VCX500; Sonics & Materials, Inc., Newtown, CT, USA) on an ice bed. After sonication, recombinant tag-free FlaB was purified using chitin-based affinity column chromatography and 50 mM 1,4-dithiothreitol solution, and the purity of the recombinant FlaB was confirmed by SDS-PAGE and Western blotting with the rabbit anti-FlaB antibody induced by glutathione *S*-transferase (GST)-FlaB. Contaminating lipopolysaccharide (LPS) was removed using an Affinity Pak Detoxi Gel Endotoxin Removing Gel (Pierce Biotechnology, Inc., Rockford, IL, USA), and the residual LPS content was determined by using a gel-clotting Endosafe LAL Kit (Charles River Endosafe, Charleston, SC, USA). The LPS levels in the flagellin preparation were kept below 0.48 EU/mL, which corresponds to 0.0096 EU per dose. The protein was suspended in sterile PBS at appropriate concentrations.

### 2.4. Tumor Implantation and Antitumor Therapy

An inoculum of 5 × 10^5^ B16-F10 tumor cells in 100 μL sterile PBS (primary tumor) was injected subcutaneously (s.c.) into the flank of C57BL/6J mice. Two days after the primary tumor cell implantation, the contralateral flank was injected s.c. with 1 × 10^5^ B16-F10 cells (abscopal tumor). When the primary tumor diameter reached approximately 5 mm, the tumor-bearing mice were randomly assigned into different treatment groups (PBS, PDT, FlaB-Vax, and PDT + FlaB-Vax, *n* = 5 per each group). We defined the day of PDT treatment as “day 0” in all experiments and in all figures (Figure 1B). The vaccine, composed of 4 µg of FlaB and 50 μg each of Tyrp1, Trp2, and gp100 peptides, was peritumorally administered 3 days before and 3 and 6 days after PDT. For PDT, mice were intravenously injected with Lipo-PhA (5 mg/kg) solution 1 day before laser irradiation. The PDT and PDT + FlaB-Vax groups received light irradiation (671 nm wavelength, 200 mW/cm) for 15 min at 24 h postinjection of Lipo-PhA. The tumor volume and body weight were measured in appropriate time interval. The tumor volume was calculated with the following formula: V = (tumor length) × (tumor width) × (tumor height)/2. For experiments exploring combinations with ICI therapy, anti-PD1 (clone RMP1-14, BioXCell, Lebanon, USA) was administered intraperitoneally (i.p.) at 200 μg per dose 4, 7, and 10 days after PDT. Mice are euthanized when the tumor volume reached 1200 mm^3^. All experiments were performed at least in duplicates and representative data are used for analyses.

### 2.5. Ethics Statement

All animal experimental procedures were performed under the approval from the Chonnam National University Institutional Animal Care and Use Committee in accordance with the protocol CNU IACUC-H-2018-66. Animal research facility maintenance and experimental procedures were carried out strictly keeping the guideline of the Animal Welfare Act legislated by the Korean Ministry of Agriculture, Food and Rural Affairs.

### 2.6. ELISpot Assay

Single-cell suspensions from spleen and TDLNs were prepared on day 7 after all treatments. Specifically, 1 × 10^6^ cells per well were seeded into Millipore ELISpot plates and stimulated with 2 µg/mL peptides. After 24-h culture, IFNγ-producing T cells were detected in accordance with the manufacturer’s instructions (R&D systems, Inc., Minneapolis, MN, USA).

### 2.7. Flow Cytometry

To detect CD8^+^ T cells that produced the tumor antigen-specific IFNγ, lymph nodes and spleens were resected from tumor-bearing mice on day 7 after all treatments and mechanically disrupted to generate single-cell suspensions. The peptides used for restimulation were 10 μg/mL of the relevant antigens: Trp2_180–188_ (SVYDFFVWL), Tyrp1_455–463_ (TAPDNLGYA), or gp100_25–33_ (EGSRNQDWL). Tumor-infiltrating lymphocytes (TILs) were analyzed as previously described [29]. Briefly, tumors were resected, weighed, digested with collagenase and DNase I in the culture medium at 37 °C for 20 min, and mechanically disrupted to generate single-cell suspensions. TILs were separated using a CD45 MACS-bead isolation protocol according to the manufacturer’s instructions (130-045-801, Miltenyi Biotec, Bergisch Gladbach, Germany). Cells were stained to identify TILs and quantified using flow cytometry. The expression of CD4, CD8 (BD Biosciences), CD11C, CXCL10, or PD-1 was examined by multiparameter flow cytometry (all antibodies from eBioscience unless stated otherwise). Cells were analyzed using a BD FACS Canto flow cytometer, and data were analyzed using the FlowJo software.

### 2.8. Depletion of CD4^+^ or CD8^+^ T Cells

Cellular subsets were depleted by administering i.p. 100 μg of depleting antibody 2 days before each treatment [22]. CD8^+^ and CD4^+^ T cells were depleted with anti-CD8α (clone 2.43, BioXCell) and anti-CD4 (clone GK1.5, BioXCell, Lebanon, USA), respectively. Depletions of CD8^+^ and CD4^+^ T cells were confirmed by flow cytometry of PBMCs.

### 2.9. Statistical Analysis

Data analysis was carried out using GraphPad Prism 8. The results are expressed as the mean ± SEM unless otherwise noted. Data were analyzed for significance using one-way ANOVA with Tukey’s test for multiple comparisons or two-way ANOVA with Bonferroni post hoc test. For the comparison of survival, the Kaplan–Meier analysis was employed. *p* values < 0.05 were considered statistically significant.

## 3. Results

### 3.1. Synthesis and Characterization of Lipo-PhA Nanoparticles and Antitumor Effects of PDT

Simple liposome and complex liposome loaded with PhA (Lipo-PhA) were synthesized by the lipid film hydration method followed by extrusion (Figure 1A and Supplementary Figure S1). The field-emission transmission electron microscopy (FE-TEM) images illustrated that the synthesized liposomes were uniform and well dispersed with *ca* 110 nm diameter (Figure 1A and Supplementary Figure S1A). PhA was loaded into liposomes (Lipo-PhA) through encapsulating into the membrane compartment of liposomes. The FE-TEM images of Lipo-PhA showed an approximate size of 130 nm (Supplementary Figure S1A). The average diameters measured by the dynamic light scattering (DLS) analysis for Lipo-PhA were 160 ± 4 and 151 ± 9 nm respectively (Supplementary Figure S1B). Zeta potentials were −4 ± 0.2 and −7 ± 3 mV, respectively, for liposome and Lipo-PhA (Supplementary Figure S1C). Liposomes bearing PhA typically in sizes between 100 and 200 nm with negative zeta potential are regarded to possess physicochemical properties for enhanced systemic circulation [30]. PhA loading into liposomes was confirmed by UV–VIS spectrophotometry, demonstrating a characteristic peak of PhA at 674 nm (Supplementary Figure S1D). Photosensitizer uptake in established tumors was proved by intravenously injecting Lipo-PhA into mice bearing subcutaneous B16-F10 tumors, which accumulated in the tumor area after 2~24 h. To analyze whether this photosensitizer accumulation is sufficient for photodynamic ablation, growing B16-F10 tumors with a diameter of 5 mm were irradiated with a focused laser beam 24 h after the Lipo-PhA injection. The PDT treatment resulted in significant ablation of primary tumor and delay in tumor growth (Figure 1C). Contralateral tumor growth in the PDT group appeared delayed, though statistical significance was not noted.

### 3.2. Cooperative Antitumor Effect of PDT and FlaB-Vax Combination on Abscopal Tumor Suppression

The strong tumor ablation and possible beneficial immunologic effects of Lipo-PhA-based PDT, suggested by a growth delay of abscopal tumors, made us consider a combinatorial approach employing immunotherapies. We hypothesized that antitumor immune responses will be enhanced by prime-boost TAA vaccinations before and after PDT. If the PDT induces immunogenic cell death and tumor antigen presentation in the TME as well as in TDLNs, the combination of PDT and specific TAA vaccination should cooperatively enhance host immune responses against the tumor. We have previously observed that the *V. vulnificus* FlaB-adjuvanted peptide cancer vaccine induced efficient therapeutic immune responses through activating antigen-specific CMI [22]. Based upon our previous studies, we combined PDT with FlaB-adjuvanted peptide vaccine (FlaB-Vax) using the schedule described in Figure 1B. To evaluate the immune abscopal effects, one more tumor was generated 2 days after the primary tumor implantation with 1/5 inoculum size at the contralateral flank. PDT and peritumoral vaccination were given to the primary tumor, and tumor growth was observed in both sides. As for the tumor burden in the primary tumor site, PDT effectively ablated tumor lump at the irradiation site, whereas combination with FlaB-Vax did not exert additional ablative effect (Figure 1C). When mice were observed until day 9, a significant growth suppression in abscopal tumors was observed in PDT, Vax, and PDT + Vax groups, where the PDT + Vax combination appeared to be most potent (Figure 1D). These results suggest that PDT may have induced immunogenic cell death liberating tumor-specific antigens at the irradiation site, but the statistical significance disappeared at day 13 suggesting that the immunity induced by PDT should have been less potent or relatively short-lived compared with Vax only treatment. The Vax only group maintained statistical significance in tumor volume reduction compared with the cancer only control group until the last day of observation. The PDT + Vax combination induced significantly enhanced abscopal immunity suppressing tumor growth in the distant site (Figure 1D). These results suggest that the combination of PDT and FlaB-Vax exerted a cooperative effect on inducing tumor-specific immune responses, which may possibly be further potentiated by ICI therapy.

### 3.3. PDT and FlaB-Vax Combination Generates Antigen-Specific IFNγ-Secretions

**Figure 2:**
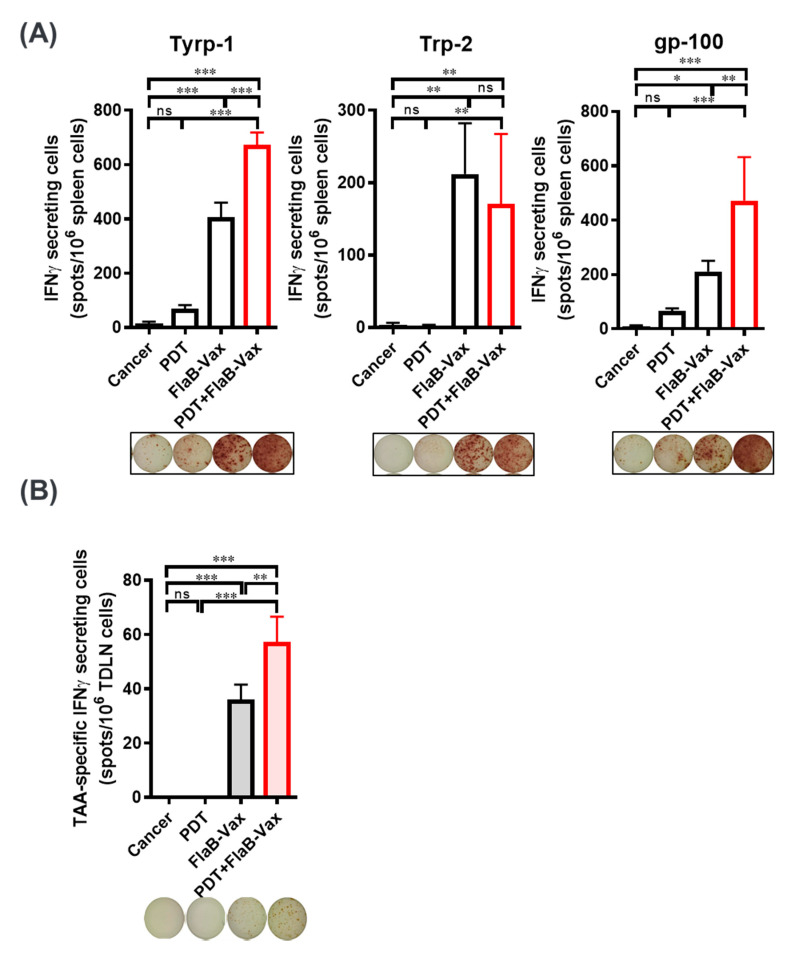
The combination of PDT and FlaB-Vax generates IFNγ-secreting antigen specific immune responses. Splenocytes (**A**) and tumor-draining lymph nodes (TDLNs) (**B**) were isolated from mice at day 7 after treatment, and then, the cells were stimulated with Tyrp1/Trp2/gp100 peptides to assess T cells secreting interferon γ (IFNγ) by ELISpots. Splenocytes and TDLN cells were prepared from 4 and 3 animals, respectively. TDLN cells are stimulated by the mixture of three peptide antigens. The results are presented as the mean ± SEM (*n* = 5 per group). *, *p* < 0.05; **, *p* < 0.01; and ***, *p* < 0.001.

The TLR5 agonist flagellins serve safe, effective, and broadly applicable immunotherapeutic agent that would enhance specific immune responses against coadministered antigens [31]. The TLR5 agonist entolimod was reported to induce CD8^+^ T cell responses through NK-dendritic cell axis within the liver, which resulted in a potent inhibition of liver metastasis of colon and mammary cancers [32]. Our group also had shown that flagellin-adjuvanted peptide vaccines induced efficacious antitumor cytotoxic T lymphocyte (CTL) responses using TC-1 murine cervical cancer models [22,23]. To specifically induce TAA-specific T cell responses, we peritumorally administered a FlaB-adjuvanted vaccine composed of triple peptide antigens (Tyrp1/Trp2/gp100) three times (Figure 1A). After the vaccination, antigen-specific T cell responses in splenocytes and TDLN cells were evaluated using IFNγ ELISPOT. The FlaB-adjuvanted vaccine (FlaB-Vax) was effective in inducing antigen-specific IFNγ-secretions systemically (splenocytes) and locally (TDLN cells) (Figure 2). The PDT + FlaB-Vax combination resulted in further enhanced levels of IFNγ-secretions after stimulation with each peptide (Tyrp1/Trp2/gp100) (Figure 2).

### 3.4. Enhanced Infiltration of Effector Memory CD8^+^ T Cells and Cross-Presenting DCs after PDT + FlaB-Vax Combination Therapy

**Figure 3:**
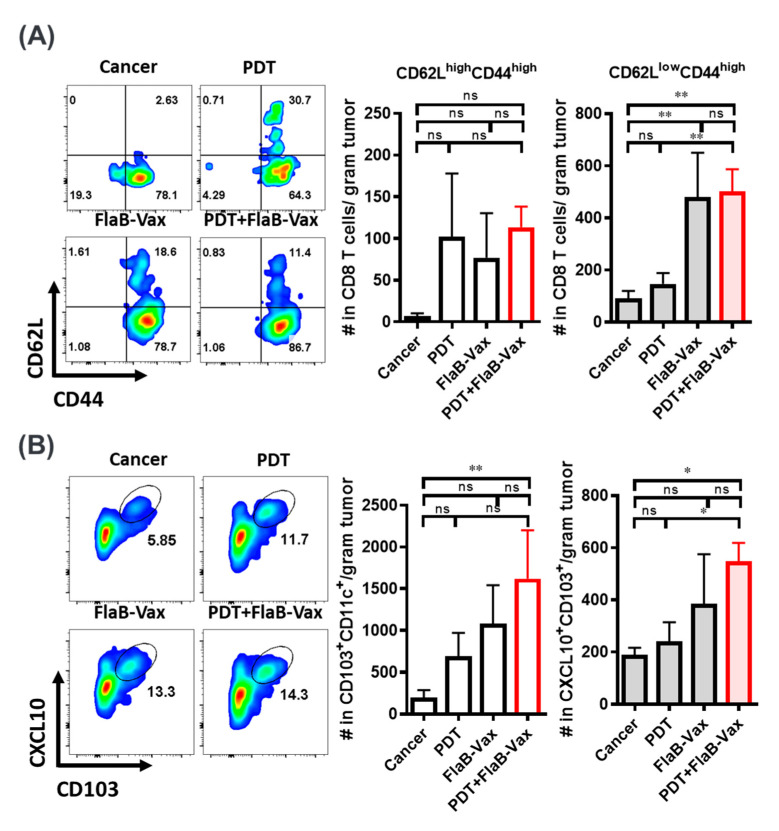
Increase in tumor-infiltrating memory CD8^+^ T cells and CD103^+^ dendritic cells (DCs) producing CXCL10 after treatments. Seven days after completion of all treatments of B16-F10-bearing mice, tumor-infiltrating lymphocytes (TILs) were prepared as a single-cell suspension with CD45^+^ MACS beads. (**A**) Representative flow plots and numbers of cells per gram of tumors of CD44 by CD62L expression on CD8^+^ T cells in TILs. (**B**) Representative flow plots and cell numbers per gram of tumors of CXCL10^+^, CD103^+^, and CD11C^+^ DCs. The results are presented as the mean ± SEM (*n* = 5 per group). *, *p* < 0.05; and **, *p* < 0.01.

Previously, although the evidence of increased T cell infiltration was noted after PDT, only the combination of PDT and immunotherapy was addressed to provide long-term disease control at local and distant tumor sites [33]. We analyzed CD8^+^ TILs by the expression of CD44 and CD62L to assume how much effector memory T cells were infiltrating in the TME. TILs were prepared from the four different groups of mice 7 days after the last treatment. Significant number of CD44^low^ CD62^low^ T cells were noted in nontreated cancer tissue (Figure 3A). In other groups, TILs appeared to be activated having CD44^high^ phenotypes. In PDT, FlaB-Vax, and PDT + FlaB-Vax groups, the infiltration of CD44^high^ and CD62L^high^ central memory T cells was noted (PDT, 102.17 ± 43.75; FlaB-Vax, 77.09 ± 30.69; FlaB-Vax, 113.61 ± 14.12 cells per gram tumor) in the TME compared with nontreated cancer-bearing group (7.31 ± 3.26 per gram tumor). As for the CD44^high^ CD62L^low^ effector/effector memory T cells, significantly increased infiltration was noted only in FlaB-Vax and PDT + FlaB-Vax combination groups suggesting a dominant role of vaccination in stimulating CD44^high^ and CD62L^low^ CD8^+^ T cell infiltration in TME (Figure 3A,B). These results indicate that PDT or FlaB-Vax should be able to induce tumor antigen-specific immune responses accompanying memory generation, which was most effective when those two treatment modalities were combined.

PDT and vaccination appeared to make the TME more T cell inflamed. A critical process in T cell priming against tumor antigens involves the recruitment and activation of Batf3-lineage dendritic cells (DCs) expressing CD103 in the mouse [34]. The CD103^+^ Batf3-lineage DCs recruit and activate T cells through CXCR3/CXCL9/CXCL10 axis via STING and type I IFN signaling [35]. The Batf3-lineage DCs are known to play important roles in inducing CTLs through antigen cross-presentation [36]. In this regard, we checked whether PDT and/or FlaB-Vax treatments employed the CD103^+^ DC-CXCL9/10 axis in recruiting T cell to the TME. We have observed that CXCL10 secreting CD103^+^ DC increased in the tumor tissue in an ascending order of nontreated cancer, PDT, FlaB-Vax, and PDT + FlaB-Vax treatments (Figure 3B). However, the gradient was not so notable in the TDLNs, while the difference between treated and nontreated groups was statistically significant (Supplementary Figure S2). For the recruitment of cross-presenting DCs to the TME, PDT and vaccination appeared to exert cooperative effects. Indeed, the highest levels of CXCL10 were observed in CD103^+^ DCs from the PDT + FlaB-Vax-treated tumors (Figure 3B). Tumor cells emigrate from primary tumor sites to draining lymph nodes via afferent lymphatic vessels and interact with immune cells there [37]. Mirroring the TME, TDLNs harbored enhanced levels of peptide-specific IFNγ-secreting T cells and CXCL10-secreting CD103^+^ cells in the combination group (Supplementary Figure S2). These results suggest that the tumor-specific CD8^+^ T cell infiltration in TME and TDLNs would have been mediated by CXCL10-secreting CD103^+^ DCs, which should have induced tumor antigen-specific CD8^+^ CTLs through tumor antigen cross-presentation.

### 3.5. CD8^+^ T Cells Mediate the Antitumor Effects of PDT + FlaB-Vax Combination Therapy

**Figure 4:**
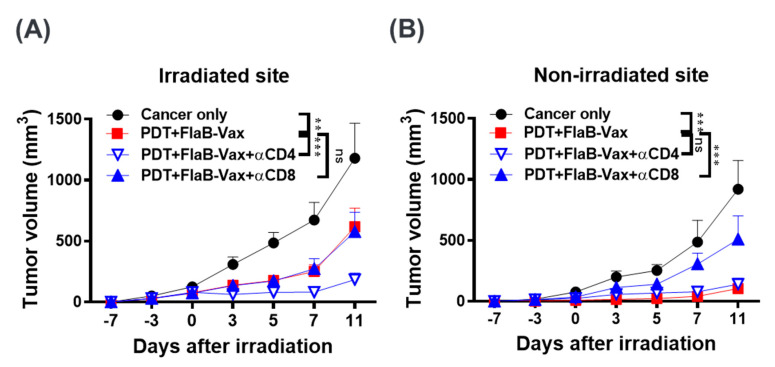
The tumor suppression effect of combination treatment was dependent on the treatment-induced systemic CD8^+^ T cell response. Depleting antibody was administered i.p. 2 days before FlaB-Vax or PDT treatment, resulting in >98% depletion of CD4^+^ or CD8^+^ cells in the blood within 24 h after injection. Irradiated (**A**) and nonirradiated abscopal tumor growth (**B**) of B16-F10 tumor-bearing mice treated with PDT and FlaB-Vax after antibody-mediated depletion of CD4^+^ or CD8^+^ T cells (*n* = 5 per group). Statistical significance was calculated by two-way ANOVA and Tukey’s multiple comparison test. **, *p* < 0.01; and ***, *p* < 0.001.

Flagellin adjuvant with peptide-based vaccine formulations is recognized by TLR5-expressing CD11c^+^ DCs, which are subsequently activated to stimulate T lymphocytes [38]. Activated CD4^+^ T lymphocytes play important roles in inducing antigen-specific T and B cell responses. TLR5 binding by flagellin was reported to activate APCs that preferentially induce Th1 responses [39]. In peptide-based cervical cancer vaccines, the antitumor efficacy was mainly mediated by CD8^+^ T cells, and the effect was dependent on IFNγ responses [22]. In the present study, the combination of PDT and FlaB-Vax induced systemic tumor-specific CD8^+^ T cell responses in lymph nodes and spleen of bilateral B16-F10 melanoma-bearing mice. Moreover, the effector memory CD8^+^ T cells were significantly induced by the combination therapy (Figure 3A). In this context, we evaluated the contribution of CD4^+^ or CD8^+^ T cells in the PDT + FlaB-Vax combination efficacy by depleting CD8^+^ or CD4^+^ T cells. Consistent with previous results [29], depletion of CD4^+^ T cells paradoxically enhanced the therapeutic effect of combination therapy in local tumor control (Figure 4A,B), whereas depletion of CD8^+^ T cells abolished the therapeutic effects of the combination therapy in abscopal tumors (Figure 4B). In the primary tumor site, CD4^+^ T cell-depleted animals showed almost completely suppressed residual tumor growth, whereas nondepleted PDT + FlaB-Vax-treated animals manifested tumor regrowth from day 7 (Figure 4A). This result clearly indicates that the abscopal responses induced by the combination treatment were almost entirely dependent on CD8^+^ CTLs, and a CD4^+^ T cell subpopulation concurrently induced by the combination treatment might have exerted interfering activities on the CTL-mediated tumor suppression. This result suggested that selective suppression of those interfering CD4^+^ T cell subpopulation would further improve the therapeutic outcome of PDT + FlaB-Vax combination treatments.

### 3.6. PD-1 Blockade Enhanced the Therapeutic Efficacy of PDT + FlaB-Vax Combination Therapy

**Figure 5:**
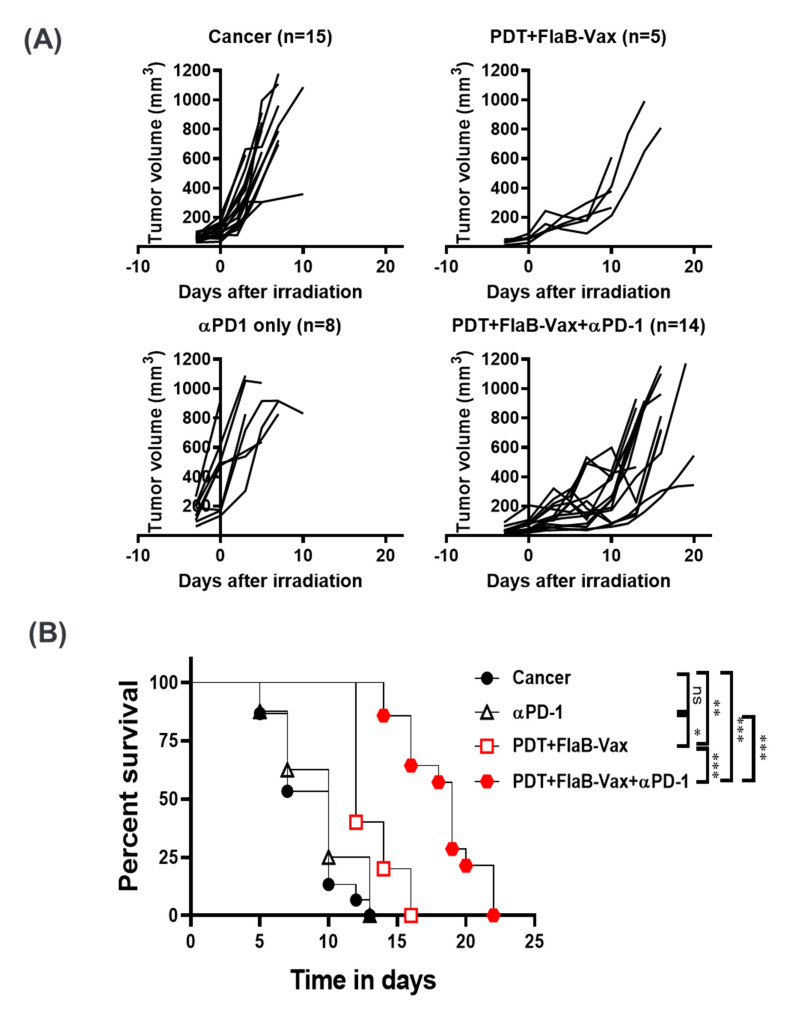

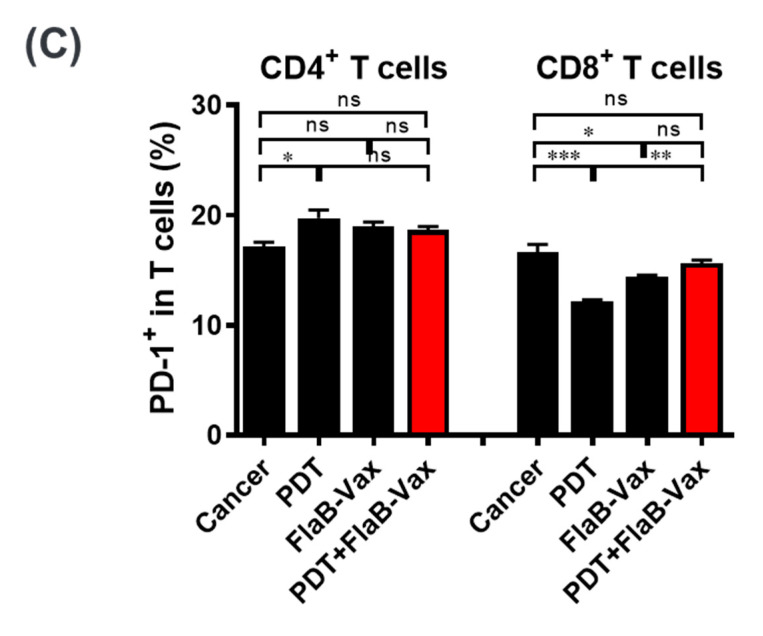
PD-1 blockade enhances the therapeutic efficacy of PDT + FlaB-Vax combination therapy. PDT and FlaB-Vax were combined with αPD-1 or isotype control antibodies (200 μg/injection) that were administered 4, 7, and 10 days following PDT. A group of mice receiving the αPD-1 mAb without the combination treatment was also included. Tumor growth control (**A**) and survival (**B**) after treatments. PD-1-expressing cells in the spleen were determined (*n* = 5 per group) (**C**). Statistical significance was calculated by one-way ANOVA for tumor growth and by Kaplan–Meier analysis for survival. *, *p* < 0.05; **, *p* < 0.01; and ***, *p* < 0.001.

T cell activation correlates with PD-1 expression, and IFNγ serves a potent signal for the induction of PD-L1 in immune and tumor cells [40]. Given the activation status and enhanced IFNγ secretion by the combined therapy, we speculated that PD-1 blockade should further potentiate the therapeutic efficacy. In this context, we further evaluated whether PD-1 blockade can enhance the synergistic antitumor effects of PDT + FlaB-Vax combination therapy. The survival of PDT + FlaB-Vax-treated animals was significantly improved by the addition of αPD-1 (Figure 5). The PDT + FlaB-Vax treatment effectively suppressed tumor growth with modest extension of survival (*p* < 0.001; cancer only vs. PDT + FlaB-Vax + αPD-1). The PD-1 blockade showed a significant effect on the survival extension of the PDT + FlaB-Vax combination treatment (Figure 5). We found that PD-1 expression on CD4^+^ T cells was significantly increased in the PDT and FlaB-Vax groups compared to the untreated group (Figure 5C). On the other hand, the PD-1 expression on CD8^+^ T cells decreased by either PDT or vaccination. However, the PDT + FlaB-Vax combination elevated the PD-1 expression to the level in nontreated cancer-bearing animals (Figure 5C). Preferential higher expression of PD-1 in treated animals may partially explain why CD4^+^ cell depletion resulted in more enhanced tumor suppression than the isotype control treatment (Figure 4A), since PD-1 expressing CD4^+^ T cells might have contributed to the immunosuppressive status in the TME/TDLNs and systemic immune compartment. These data mechanistically address how the therapeutic efficacy of PDT + FlaB-Vax combination against B16-F10 melanoma tumors was potentiated by the PD-1 blockade. Even in the PDT + FlaB-Vax combination group additionally treated with αPD-1, the tumor began to regrow after a while (Figure 5A). This result suggested that the αPD-1-mediated tumor suppression may not be an irreversible therapeutic outcome, which is dependent upon the half-life of the antibody medicine.

## 4. Discussion

ICI therapy drastically changed the landscape in cancer therapy. Almost every type of cancers was tried by ICI therapy using diverse agents from different manufacturers. More importantly, ICI approvals for cancer treatments are ever expanding. However, the majority of patients still do not demonstrate a durable long-term response following ICI therapies. Even so, ICI therapy serves the linchpin in the modern cancer immunotherapy [41]. To improve the efficacy of ICI therapies, there are ongoing efforts combining with other therapeutic modalities to achieve synergistic effects. The current ICIs (anti-PD-1/PD-L1 and anti-CTLA-4) are combined with newer agents blocking other novel checkpoints (TIM-3, LAG-3, VISTA, TIGIT, and others), immunotherapies (adoptive cell therapy, CAR-T therapy, cancer vaccines, and cytokines), and delivery strategies (bispecific antibodies and other delivery platforms). Combinations with traditional cancer therapies such as radiation therapy and chemotherapy inducing immunogenic cell deaths brought excellent therapeutic outcomes also [42]. ICI therapies result in durable responses in only 20–40% of patients. Clinical evidence discloses that a substantial number of initial responders will ultimately relapse with lethal, drug-resistant diseases in months or years later. Understanding of the underlying mechanisms of primary and acquired resistance to ICI therapies [42,43] would enable immunotherapy to be more successful treatment options for wider range of cancer types. Combinatorial approaches should target those resistance mechanisms. In the present study, we show that PDT would induce immunogenic cell death generating abscopal tumor suppression and a flagellin-adjuvanted peptide vaccine formulation induced TAA-specific immune responses resulting in TIL infiltration, which should be efficaciously combined with anti-PD1 ICI therapy.

PDT has the advantages of precisely killing tumors with low toxicity, easy combination with other primary or adjuvant therapies, repeatability, and improvements in quality of life [44]. Due to their amphiphilic characteristics, liposomes have proven suitable carriers for improving the photophysical properties of photosensitizers. Moreover, liposomes improve the efficiency and safety of PDT through extended permeation and retention in the TME [45]. PhA is a photosensitive chlorophyll metabolite with immune stimulatory activity [46], which, at adequate concentrations, activates the cytotoxic T cell immune responses against tumor cells. Therefore, liposomes with PhA have been applied as an active therapeutic component in anticancer therapies. The combination of intratumoral injection of DCs and local PDT resulted in a striking antitumor effect with potent systemic antitumor immunity [47]. In this study, the potential of PDT as a component of immune combination therapy was corroborated using bilateral highly aggressive B16-F10 melanoma “cold tumor” model. We observed that PDT in combination with FlaB-Vax had significant growth suppression effects on both local and distant tumors. Peptide-based FlaB-Vax significantly induced systemic immune responses secreting IFNγ as well as antigen cross-presenting DCs in TME and draining LNs.

Because of the infrequency and generally unsatisfactory nature of immune response after PDT, the administration of an immune stimulant or adjuvant in combination with PDT has been recommended to increase the frequency or strength of antitumor immune responses [48]. In a primary renal tumor animal model, which is known to be rather responsive to immunotherapies, combined PDT and PD-1/PD-L1 blockade resulted in regression of primary tumors, prevented growth of lung metastases, and prolonged survival, but neither treatment alone showed sufficient antitumor effects [49]. PDT combined with synthetic long peptide vaccination induced primary tumor rejection with tumor specific CD8^+^ T cells response in TC-1 established tumor model [50]. We have also shown that intravaginal coadministration of the E6/E7 peptides with flagellin resulted in tumor suppression and long-term survival of tumor-bearing mice [23]. We hypothesized that the TLR5 agonist flagellin, which is a stable protein being resilient to physicochemical insults, will potentiate peptide vaccines and survive physicochemically hostile tumor microenvironment induced by PDT. Since flagellin is a very stable bacterial protein that automatically assemble to form the locomotive apparatus flagellum, any protein or polypeptide antigens directly fused to flagellin makes very stable vaccines as well [51,52], which presents a superior platform for generating clinically applicable pharmaceutical products. Flagellin is highly resistant to high temperature, redox variation, and other physicochemical challenges in body fluids [28]. To induce tumor-specific MHC class I restricted CTL responses, we employed multivalent short peptide vaccine formulation using a flagellin as an adjuvant. Since pre-existing PD-1-expressing TILs are important requisite for better responses in anti-PD-1 ICI therapies, we administered anti-PD-1 ICI after vaccination that would activate tumor-specific T cell responses and PD-1 expression in TILs. Although PDT did not influence TIL infiltration, the FlaB-Vax and PDT + FlaB-Vax groups showed significant increase in the effector/effector memory CD8^+^ T cells in the TME, which would be further invigorated by the anti-PD-1 ICI treatment. The FlaB-Vax should have optimally primed and tuned antitumor CD8^+^ T cells to be responsive to the anti-PD-1 therapy [17]. Given that IFNγ has dual roles in tumorimmunity; one as a hallmark of antitumor immune response and the other as an inducer of PD-L1 expression [53], the combination of PDT + FlaB-Vax with PD-1 blockade might have exerted an additional role of breaking the negative feedback of IFNγ-mediated PD-L1 expression. Notably, the depletion of CD4^+^ cells significantly enhanced the effect of PDT + FlaB-Vax in local tumor control (Figure 4A), suggesting rather tumor-promoting effect of CD4^+^ cell population. Since Treg cells can be induced and differentiated from the traditional T cells in the TME [54], it is likely that antitumor effect of the CD4^+^ cell depletion is related with Treg generation, infiltration, and, possibly, blockade.

Among various DC subtypes, Batf3-dependent conventional DCs (cDCs) play important roles in cross-presentation of tumor cell-associated antigens to CD8^+^ T cells [55]. Localization of cDCs in tumor tissue correlates with improved infiltration of CD8^+^ T cells and tumor-specific T cell immunity. This DC subset cross-presents extracellular antigens, particularly cell-associated antigens, to CD8^+^ T cells with high efficiency [56]. In mice, CD8α^+^ DCs are natively found in lymphoid tissues, whereas CD103^+^ DCs are deployed in peripheral tissues and migrate to LNs to meet T cells for antigen presentation [55]. Recent evidence in T cell-inflamed tumors highlights the key role of CD103^+^ DCs in the baseline CD8^+^ CTL-mediated immune response against tumor antigens, and their presence in the TME of human tumors correlates with the secretion of CXCL9/10 [56]. Our data indicate that CD103^+^ DCs within the TME were potently induced by the PDT + FlaB-Vax combination treatment, thus contributing to the effector function of the antitumor T cell response. Either PDT or FlaB-Vax treatment seems to be effective in modulating TME for cDC recruitment, but sufficient activation of them inducing CXCL10 production was effectively achieved when the two treatment modalities were combined (Figure 3 and Supplementary Figure S2). These activated cDCs would effectively recruit CD8^+^ T cells into the TME through CXCL10 chemotactic signaling. Recruited CD8^+^ CTLs will be presented with tumor antigens released from dying tumor cells affected by the PDT + FlaB-Vax treatment, which should subsequently be activated by TME cytokines to express PD-1. When anti-PD-1 ICI was given at this stage, antitumor immune response might be further reinvigorated.

## 5. Conclusions

We show that the FlaB-adjuvanted therapeutic cancer vaccine can be efficiently combined with PDT to inhibit established B16-F10 tumors, being evidenced by strong abscopal tumor suppression and induction of robust systemic immune responses. Furthermore, the PDT + FlaB-Vax combination therapy effectively enhanced the PD-1 blockade. This is the first study highlighting the relevance of combining PDT and therapeutic cancer vaccines adjuvanted with TLR5 ligand to enhance immune checkpoint inhibition.

## Supplementary Materials

The following are available online at https://www.mdpi.com/2073-4409/9/11/2432/s1, Figure S1: Physicochemical characterization of liposome-pheophorbide A (Lipo-PhA) for photodynamic therapy (PDT), Figure S1: Combination therapy induced CXCL10^+^CD103^+^ DCs in tumor draining lymph nodes (TDLNs).

## Abbreviations

CMI	cell-mediated immunity
CTL	cytotoxic T lymphocyte
DAMP	damage-associated molecular pattern
DC	dendritic cell
FlaB-Vax	flagellin-adjuvanted tumor-specific peptide vaccination
ICI	immune checkpoint inhibitor
ICS	intracellular cytokine staining
Lipo-PhA	liposome-based pheophorbide A
PDT	photodynamic therapy
PhA	pheophorbide A
TAA	tumor-associated antigen
TDLN	tumor-draining lymph node
TLR	Toll-like receptor
TME	tumor microenvironment

## References

[1] Huang Y.Y., Vecchio D., Avci P., Yin R., Garcia-Diaz M., Hamblin M.R. (2013). Melanoma resistance to photodynamic therapy: New insights. Biol. Chem..

[2] Larkin J., Chiarion-Sileni V., Gonzalez R., Grob J.J., Cowey C.L., Lao C.D., Schadendorf D., Dummer R., Smylie M., Rutkowski P. (2015). Combined Nivolumab and Ipilimumab or Monotherapy in Untreated Melanoma. N. Engl. J. Med..

[3] Specenier P. (2016). Ipilimumab in melanoma. Expert. Rev. Anticancer.

[4] Robert C., Schachter J., Long G.V., Arance A., Grob J.J., Mortier L., Daud A., Carlino M.S., McNeil C., Lotem M. (2015). Pembrolizumab versus Ipilimumab in Advanced Melanoma. N. Engl. J. Med..

[5] Baldea I., Giurgiu L., Teacoe I.D., Olteanu D.E., Olteanu F.C., Clichici S., Filip G.A. (2018). Photodynamic Therapy in Melanoma—Where do we Stand?. Curr. Med. Chem..

[6] Celli J.P., Spring B.Q., Rizvi I., Evans C.L., Samkoe K.S., Verma S., Pogue B.W., Hasan T. (2010). Imaging and photodynamic therapy: Mechanisms, monitoring, and optimization. Chem. Rev..

[7] Kleinovink J.W., Fransen M.F., Lowik C.W., Ossendorp F. (2017). Photodynamic-Immune Checkpoint Therapy Eradicates Local and Distant Tumors by CD8(+) T Cells. Cancer Immunol. Res..

[8] Castano A.P., Mroz P., Hamblin M.R. (2006). Photodynamic therapy and anti-tumour immunity. Nat. Rev. Cancer.

[9] Li X., Lovell J.F., Yoon J., Chen X. (2020). Clinical development and potential of photothermal and photodynamic therapies for cancer. Nat. Rev. Clin. Oncol..

[10] Siegel R.L., Miller K.D., Jemal A. (2016). Cancer statistics, 2016. CA Cancer J. Clin..

[11] Robert C., Thomas L., Bondarenko I., O’Day S., Weber J., Garbe C., Lebbe C., Baurain J.F., Testori A., Grob J.J. (2011). Ipilimumab plus dacarbazine for previously untreated metastatic melanoma. N. Engl. J. Med..

[12] Robert C., Long G.V., Brady B., Dutriaux C., Maio M., Mortier L., Hassel J.C., Rutkowski P., McNeil C., Kalinka-Warzocha E. (2015). Nivolumab in previously untreated melanoma without BRAF mutation. N. Engl. J. Med..

[13] Weber J.S., D’Angelo S.P., Minor D., Hodi F.S., Gutzmer R., Neyns B., Hoeller C., Khushalani N.I., Miller W.H., Lao C.D. (2015). Nivolumab versus chemotherapy in patients with advanced melanoma who progressed after anti-CTLA-4 treatment (CheckMate 037): A randomised, controlled, open-label, phase 3 trial. Lancet Oncol..

[14] Robert C., Ribas A., Schachter J., Arance A., Grob J.J., Mortier L., Daud A., Carlino M.S., McNeil C.M., Lotem M. (2019). Pembrolizumab versus ipilimumab in advanced melanoma (KEYNOTE-006): Post-hoc 5-year results from an open-label, multicentre, randomised, controlled, phase 3 study. Lancet Oncol..

[15] McGranahan N., Furness A.J., Rosenthal R., Ramskov S., Lyngaa R., Saini S.K., Jamal-Hanjani M., Wilson G.A., Birkbak N.J., Hiley C.T. (2016). Clonal neoantigens elicit T cell immunoreactivity and sensitivity to immune checkpoint blockade. Science.

[16] Tumeh P.C., Harview C.L., Yearley J.H., Shintaku I.P., Taylor E.J., Robert L., Chmielowski B., Spasic M., Henry G., Ciobanu V. (2014). PD-1 blockade induces responses by inhibiting adaptive immune resistance. Nature.

[17] Verma V., Shrimali R.K., Ahmad S., Dai W., Wang H., Lu S., Nandre R., Gaur P., Lopez J., Sade-Feldman M. (2019). PD-1 blockade in subprimed CD8 cells induces dysfunctional PD-1(+)CD38(hi) cells and anti-PD-1 resistance. Nat. Immunol..

[18] Ribas A., Shin D.S., Zaretsky J., Frederiksen J., Cornish A., Avramis E., Seja E., Kivork C., Siebert J., Kaplan-Lefko P. (2016). PD-1 Blockade Expands Intratumoral Memory T Cells. Cancer Immunol. Res..

[19] Grenier J.M., Yeung S.T., Khanna K.M. (2018). Combination Immunotherapy: Taking Cancer Vaccines to the Next Level. Front. Immunol..

[20] Sharma P., Allison J.P. (2015). Immune checkpoint targeting in cancer therapy: Toward combination strategies with curative potential. Cell.

[21] Durgeau A., Virk Y., Corgnac S., Mami-Chouaib F. (2018). Recent Advances in Targeting CD8 T-Cell Immunity for More Effective Cancer Immunotherapy. Front. Immunol..

[22] Nguyen C.T., Hong S.H., Sin J.I., Vu H.V., Jeong K., Cho K.O., Uematsu S., Akira S., Lee S.E., Rhee J.H. (2013). Flagellin enhances tumor-specific CD8(+) T cell immune responses through TLR5 stimulation in a therapeutic cancer vaccine model. Vaccine.

[23] Lee S.E., Hong S.H., Verma V., Lee Y.S., Duong T.N., Jeong K., Uthaman S., Sung Y.C., Lee J.T., Park I.K. (2016). Flagellin is a strong vaginal adjuvant of a therapeutic vaccine for genital cancer. Oncoimmunology.

[24] Zheng J.H., Nguyen V.H., Jiang S.N., Park S.H., Tan W., Hong S.H., Shin M.G., Chung I.J., Hong Y., Bom H.S. (2017). Two-step enhanced cancer immunotherapy with engineered Salmonella typhimurium secreting heterologous flagellin. Sci. Transl. Med..

[25] Vijayakumar G., McCroskery S., Palese P. (2020). Engineering Newcastle Disease Virus as an Oncolytic Vector for Intratumoral Delivery of Immune Checkpoint Inhibitors and Immunocytokines. J. Virol..

[26] Zhang H. (2017). Thin-Film Hydration Followed by Extrusion Method for Liposome Preparation. Methods Mol. Biol..

[27] Moynihan K.D., Opel C.F., Szeto G.L., Tzeng A., Zhu E.F., Engreitz J.M., Williams R.T., Rakhra K., Zhang M.H., Rothschilds A.M. (2016). Eradication of large established tumors in mice by combination immunotherapy that engages innate and adaptive immune responses. Nat. Med..

[28] Lee S.E., Kim S.Y., Jeong B.C., Kim Y.R., Bae S.J., Ahn O.S., Lee J.J., Song H.C., Kim J.M., Choy H.E. (2006). A bacterial flagellin, Vibrio vulnificus FlaB, has a strong mucosal adjuvant activity to induce protective immunity. Infect. Immun..

[29] Dovedi S.J., Adlard A.L., Lipowska-Bhalla G., McKenna C., Jones S., Cheadle E.J., Stratford I.J., Poon E., Morrow M., Stewart R. (2014). Acquired resistance to fractionated radiotherapy can be overcome by concurrent PD-L1 blockade. Cancer Res..

[30] Bozzuto G., Molinari A. (2015). Liposomes as nanomedical devices. Int. J. Nanomed..

[31] Burdelya L.G., Brackett C.M., Kojouharov B., Gitlin I.I., Leonova K.I., Gleiberman A.S., Aygun-Sunar S., Veith J., Johnson C., Haderski G.J. (2013). Central role of liver in anticancer and radioprotective activities of Toll-like receptor 5 agonist. Proc. Natl. Acad. Sci. USA.

[32] Brackett C.M., Kojouharov B., Veith J., Greene K.F., Burdelya L.G., Gollnick S.O., Abrams S.I., Gudkov A.V. (2016). Toll-like receptor-5 agonist, entolimod, suppresses metastasis and induces immunity by stimulating an NK-dendritic-CD8+ T-cell axis. Proc. Natl. Acad. Sci. USA.

[33] Yang Y., Hu Y., Wang H. (2016). Targeting Antitumor Immune Response for Enhancing the Efficacy of Photodynamic Therapy of Cancer: Recent Advances and Future Perspectives. Oxid. Med. Cell. Longev..

[34] Spranger S., Sivan A., Corrales L., Gajewski T.F. (2016). Tumor and Host Factors Controlling Antitumor Immunity and Efficacy of Cancer Immunotherapy. Adv. Immunol..

[35] Woo S.R., Fuertes M.B., Corrales L., Spranger S., Furdyna M.J., Leung M.Y., Duggan R., Wang Y., Barber G.N., Fitzgerald K.A. (2014). STING-dependent cytosolic DNA sensing mediates innate immune recognition of immunogenic tumors. Immunity.

[36] Hildner K., Edelson B.T., Purtha W.E., Diamond M., Matsushita H., Kohyama M., Calderon B., Schraml B.U., Unanue E.R., Diamond M.S. (2008). Batf3 deficiency reveals a critical role for CD8alpha+ dendritic cells in cytotoxic T cell immunity. Science.

[37] Torcellan T., Hampton H.R., Bailey J., Tomura M., Brink R., Chtanova T. (2017). In vivo photolabeling of tumor-infiltrating cells reveals highly regulated egress of T-cell subsets from tumors. Proc. Natl. Acad. Sci. USA.

[38] Mizel S.B., Bates J.T. (2010). Flagellin as an adjuvant: Cellular mechanisms and potential. J. Immunol..

[39] Ding X., Bian G., Leigh N.D., Qiu J., McCarthy P.L., Liu H., Aygun-Sunar S., Burdelya L.G., Gudkov A.V., Cao X. (2012). A TLR5 agonist enhances CD8(+) T cell-mediated graft-versus-tumor effect without exacerbating graft-versus-host disease. J. Immunol..

[40] Chamoto K., Al-Habsi M., Honjo T. (2017). Role of PD-1 in Immunity and Diseases. Curr. Top. Microbiol. Immunol..

[41] Wilky B.A. (2019). Immune checkpoint inhibitors: The linchpins of modern immunotherapy. Immunol. Rev..

[42] Kon E., Benhar I. (2019). Immune checkpoint inhibitor combinations: Current efforts and important aspects for success. Drug Res. Updates.

[43] Syn N.L., Teng M.W.L., Mok T.S.K., Soo R.A. (2017). De-novo and acquired resistance to immune checkpoint targeting. Lancet Oncol..

[44] Hwang H.S., Shin H., Han J., Na K. (2018). Combination of photodynamic therapy (PDT) and anti-tumor immunity in cancer therapy. J. Pharm. Investig..

[45] Muehlmann L.A., Joanitti G.A., Silva J.R., Longo J.P., Azevedo R.B. (2011). Liposomal photosensitizers: Potential platforms for anticancer photodynamic therapy. Braz. J. Med. Biol. Res..

[46] Bui-Xuan N.H., Tang P.M., Wong C.K., Chan J.Y., Cheung K.K., Jiang J.L., Fung K.P. (2011). Pheophorbide a: A photosensitizer with immunostimulating activities on mouse macrophage RAW 264.7 cells in the absence of irradiation. Cell Immunol..

[47] Saji H., Song W., Furumoto K., Kato H., Engleman E.G. (2006). Systemic antitumor effect of intratumoral injection of dendritic cells in combination with local photodynamic therapy. Clin. Cancer Res..

[48] Mroz P., Hashmi J.T., Huang Y.Y., Lange N., Hamblin M.R. (2011). Stimulation of anti-tumor immunity by photodynamic therapy. Expert Rev. Clin. Immunol..

[49] O’Shaughnessy M.J., Murray K.S., La Rosa S.P., Budhu S., Merghoub T., Somma A., Monette S., Kim K., Corradi R.B., Scherz A. (2018). Systemic Antitumor Immunity by PD-1/PD-L1 Inhibition Is Potentiated by Vascular-Targeted Photodynamic Therapy of Primary Tumors. Clin. Cancer Res..

[50] Kleinovink J.W., van Driel P.B., Snoeks T.J., Prokopi N., Fransen M.F., Cruz L.J., Mezzanotte L., Chan A., Lowik C.W., Ossendorp F. (2016). Combination of Photodynamic Therapy and Specific Immunotherapy Efficiently Eradicates Established Tumors. Clin. Cancer Res..

[51] Puth S., Hong S.H., Na H.S., Lee H.H., Lee Y.S., Kim S.Y., Tan W., Hwang H.S., Sivasamy S., Jeong K. (2019). A built-in adjuvant-engineered mucosal vaccine against dysbiotic periodontal diseases. Mucosal Immunol..

[52] Nguyen C.T., Kim S.Y., Kim M.S., Lee S.E., Rhee J.H. (2011). Intranasal immunization with recombinant PspA fused with a flagellin enhances cross-protective immunity against Streptococcus pneumoniae infection in mice. Vaccine.

[53] Mandai M., Hamanishi J., Abiko K., Matsumura N., Baba T., Konishi I. (2016). Dual Faces of IFNgamma in Cancer Progression: A Role of PD-L1 Induction in the Determination of Pro- and Antitumor Immunity. Clin. Cancer Res..

[54] Li C., Jiang P., Wei S., Xu X., Wang J. (2020). Regulatory T cells in tumor microenvironment: New mechanisms, potential therapeutic strategies and future prospects. Mol. Cancer.

[55] Sanchez-Paulete A.R., Teijeira A., Cueto F.J., Garasa S., Perez-Gracia J.L., Sanchez-Arraez A., Sancho D., Melero I. (2017). Antigen cross-presentation and T-cell cross-priming in cancer immunology and immunotherapy. Ann. Oncol. Off. J. Eur. Soc. Med. Oncol..

[56] Spranger S., Dai D., Horton B., Gajewski T.F. (2017). Tumor-Residing Batf3 Dendritic Cells Are Required for Effector T Cell Trafficking and Adoptive T Cell Therapy. Cancer Cell.

