# Supplementary material for: Combination of Photodynamic Therapy and a Flagellin-Adjuvanted Cancer Vaccine Potentiated the Anti-PD-1-Mediated Melanoma Suppression
Source: Cells. 2020 Nov 7;9(11):2432. doi: 10.3390/cells9112432 (PMC7694978; doi:10.3390/cells9112432)
Supplement: Supplementary file 1 [file cells-09-02432-s001.zip › PDT VAX new Suppl Figs 20201013.pptx]

## Slide 1
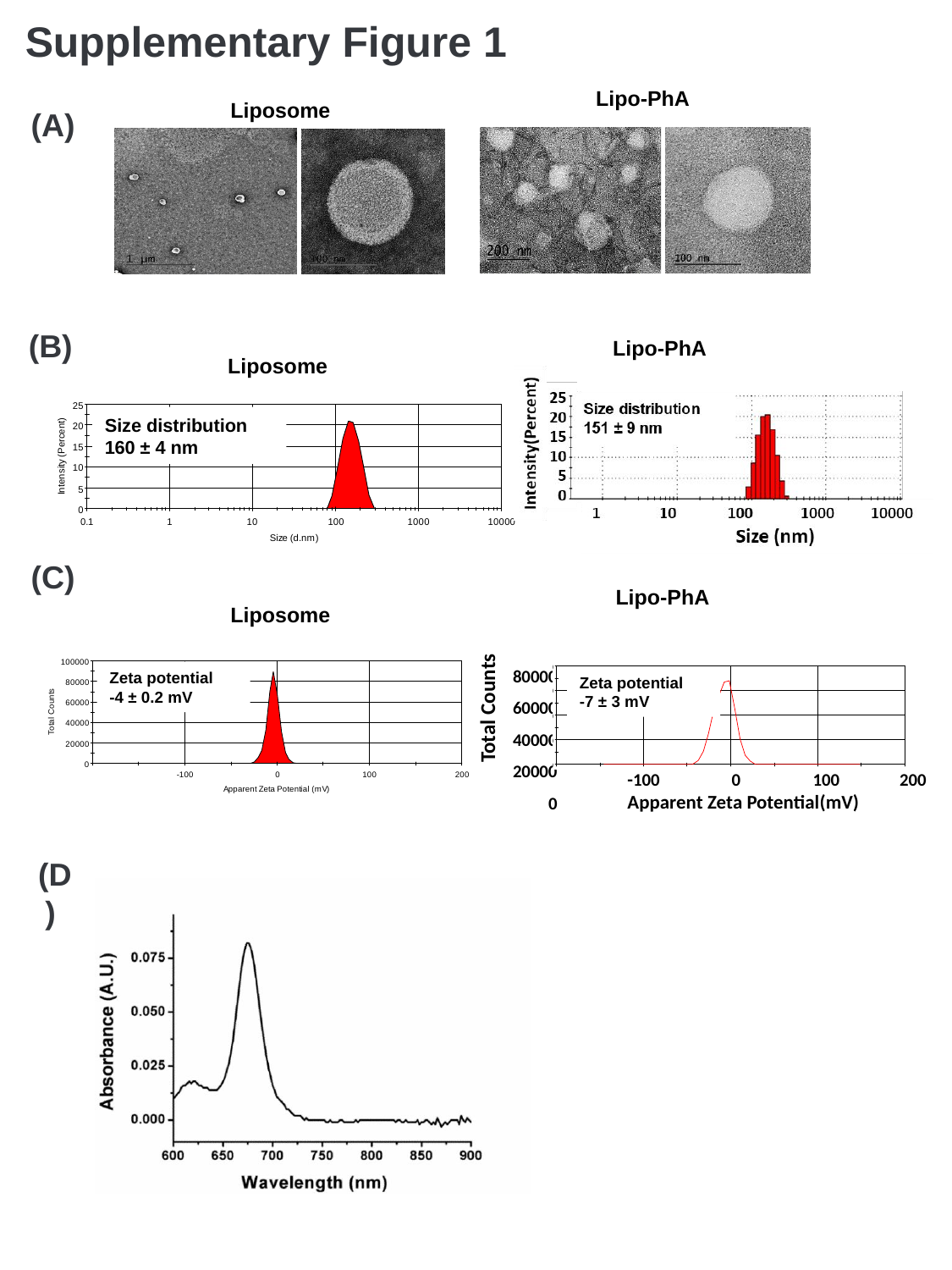

Supplementary Figure 1
Lipo-PhA
Liposome
(A)
(B)
Lipo-PhA
Liposome
Size distribution
160 ± 4 nm
(C)
Lipo-PhA
Liposome
80000
60000
40000
20000
0
Zeta potential
-7 ± 3 mV
Total Counts
-100 0 100 200
Apparent Zeta Potential(mV)
Zeta potential
-4 ± 0.2 mV
(D)

## Slide 2
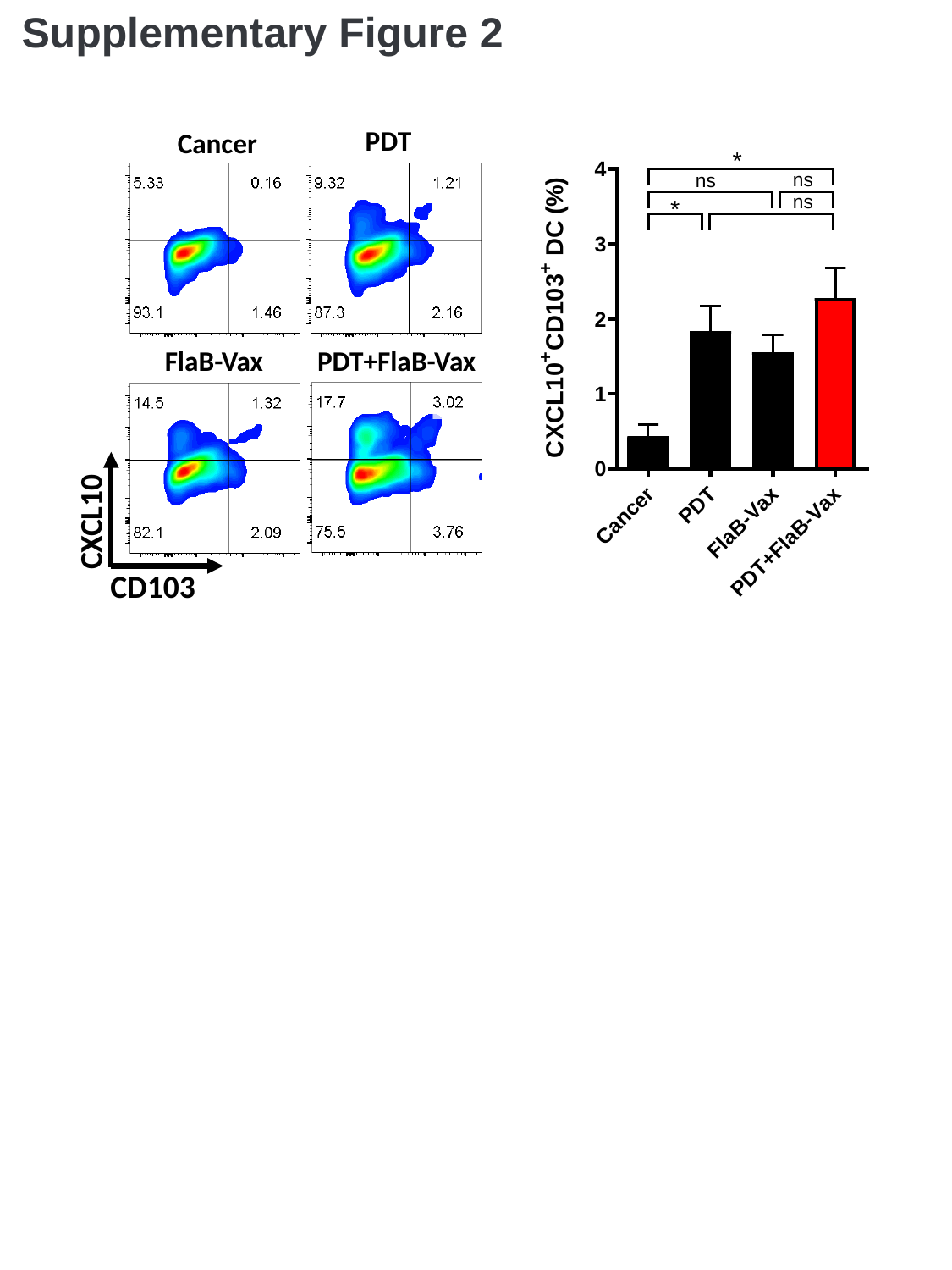

Supplementary Figure 2
PDT
Cancer
PDT+FlaB-Vax
FlaB-Vax
CXCL10
CD103
