# Supplementary material for: Combination of Photodynamic Therapy and a Flagellin-Adjuvanted Cancer Vaccine Potentiated the Anti-PD-1-Mediated Melanoma Suppression
Source: Cells. 2020 Nov 7;9(11):2432. doi: 10.3390/cells9112432 (PMC7694978; doi:10.3390/cells9112432)
Supplement: Supplementary file 1 [file cells-09-02432-s001.zip › Supplementary information Fig Legend.docx]

**Supplementary Figure S1.** **Physicochemical characterization of liposome-pheophorbide A (Lipo-PhA) for photodynamic therapy (PDT).** **(A)** Field emission transmission electron microscopy (FE-TEM) images of liposome and Lipo-PhA. Dynamic light scattering analysis determining hydrodynamic size **(B)** and zeta potential **(C)** of Lipo-PhA. **(D)** UV-Vis spectrum of Lipo-PhA demonstrating a characteristic peak at 674 nm.

**Supplementary Figure S2**. **Combination therapy induced CXCL10^+^CD103^+^ DCs in tumor draining lymph nodes (TDLNs).** Percentage of CXCL10^+^ cells gated from CD103^+^CD11c^+^ cells in TDLNs at 7 days posttreatment. The results are presented as the means ± the SEM, and statistical analysis was performed by the one-way ANOVA using the GraphPad Prism software. *, *P* < 0.05; **, *P* < 0.01; and ***, *P* < 0.001.
